# Supplementary material for: Are carbon emissions trading and green financial instruments synergistic? -Comprehensive quantitative research based on content analysis
Source: PLoS One. 2024 Mar 7;19(3):e0298601. doi: 10.1371/journal.pone.0298601 (PMC10919601; doi:10.1371/journal.pone.0298601)
Supplement: S1 Data — (ZIP) [file pone.0298601.s001.zip › Data/地级市Z_F工作报告.zip!/╜¡╦╒/┴1⁄4╘╞╕█╩╨/└┤╫╘╣1⁄2╓┌║┼í╛┬φ┐╦╩2╛▌═°í┐_2018.docx]

各位代表：

现在，我代表市人民政府向大会作工作报告，请予审议，并请各位政协委员提出意见。

2017年工作回顾

2017年是本届政府的开局之年。一年来，我们在市委坚强领导下，认真学习贯彻以习近平同志为核心的党中央治国理政新理念新思想新战略，抢抓国家“一带一路”建设和江苏沿海开发重大机遇，深化供给侧结构性改革，深入推进以港兴市、产业强市、创新驱动、绿色发展、协调共进五大战略，统筹做好改革发展稳定各项工作，较好完成了市十四届人大一次会议确定的目标任务。预计实现地区生产总值2630亿元，增长7.5%；一般公共预算收入214.8亿元，同口径增长8.4%、实际增长20.9%；固定资产投资、社会消费品零售总额分别增长10%和11.8%；居民消费价格基本稳定，城乡居民人均可支配收入分别增长8.6%、9.5%。

一年来，我市发展迈出坚实步伐，一批重要事项取得突破。中哈两国元首见证亚欧跨境运输视频连线，中国-中亚合作论坛、连云港论坛成功举办，连云港元素在“一带一路”开放大局中进一步彰显；国家级石化产业基地获批，盛虹炼化等千亿级项目加快建设，临港产业集群发展势头良好；恒瑞医药入选福布斯全球百家最具创新力公司，中复神鹰获国家科技进步一等奖，新兴产业发展前景广阔；全市上下同心同德、积极进取，高水平创成国家卫生城市，获评全国首家城市家具标准化示范城市；“现代乡贤”方敬当选全国道德模范，“直背”教师杨晓红获评中国好人，充分展示了港城人民守望相助、无私奉献的大爱情怀。

一年来，我们围绕目标狠抓落实，重点做了以下工作：

一是转型发展取得新成绩。工业经济提质增效，实现应税销售收入2366亿元，增长24.8%。“三大行动计划”深入实施，28家优强企业应税销售占全市52%，新培育科技小巨人企业21家，10家企业场外挂牌。“三新一高”产业实现产值2100亿元。亿元以上重点产业项目当年完成投资635亿元、增长45%，斯尔邦石化一期竣工投产，中化精细化工开工建设，东华能源、卫星石化成功签约。万象集团晋升建筑业特级资质，城建大厦项目获评鲁班奖。现代服务业快速发展，规模以上服务业营收增速全省第一。电子商务发展迅速，网络零售增长24%，获批省级电商示范园区3个。花果山入选全国最佳文化旅游示范景区，全市游客接待量、旅游收入分别增长15%和16%。新增金融机构6家，社会融资规模增量585亿元。市大数据中心一期工程投用，紫光大数据产业基地加快建设。农业现代化稳步推进，新增高标准农田30.4万亩，粮食丰产丰收，设施农业面积占比19.5%，新增市级龙头企业50家。完成农产品出口5亿美元。农机化综合水平达80.5%，灌南县获评全国粮食生产全程机械化示范县。

二是交汇点建设实现新突破。连盐铁路建成调试，连淮扬镇、连青铁路加快推进，连徐高铁全面开工，我市加速迈向“高铁时代”。港口完成吞吐量2.3亿吨、集装箱471万标箱，海河联运突破800万吨，集装箱铁水联运增长45.8%。国际班列运输增长60%。亚洲最大客滚船“和谐云港”成功首航。30万吨级航道二期开工建设，徐圩港区防波堤主体建成，赣榆港区10万吨级航道延伸工可获批，燕尾港口岸临时开放。中哈物流基地粮食筒仓投产运营，进出货物和集装箱分别增长32%、82%。上合物流园形成450万吨堆存能力，智慧物流中心建成投用。多式联运监管中心挂牌运行，国际贸易“单一窗口”建设成效明显，口岸通关和贸易便利化水平显著提升。空港口岸正式开放，旅客年吞吐量突破100万人次。外向型经济加快发展，实际利用外资6.7亿美元，外贸进出口总额增长16%。中国城市家具标准化国际会议、国际医药技术大会、连博会、农洽会等成功举办。

三是城乡面貌呈现新变化。推进新一轮城市总规修编，提高城市公共服务能力和均等化水平。铁路客运枢纽一期工程完工。新改建主次干道40条，新辟优化公交线路29条。新增停车泊位2200个。改造老旧小区68个，棚户区改造新开工21500套。新改建城市公园、街头游园14个，城区绿化亮化进一步提升。新改建市政管网61公里，增加管道燃气用户6500户。建成区取缔占道经营，推行垃圾分类处理，餐厨废弃物规范处置。海州水厂深度处理调试运行。建成乡镇污水处理厂5座，新改建镇村道路1000公里，农村危房改造2010户，完成农村改厕4.5万座。黄窝村成为全国美丽乡村示范村，浦南镇获批国家级美丽宜居小镇，赣榆区省级卫生乡镇全覆盖，东海县通过国家卫生县城考核。

四是改革创新迸发新活力。深化供给侧结构性改革，淘汰小型化工企业42家、冶金企业4家，化解船舶产能20万吨。商品房去库存完成省定目标。多措并举降低企业成本30亿元以上。深化财税体制改革，推行绩效评价管理。深化国企国资改革，市属监管企业主业更加突出，管理更加规范。行政审批“3550”改革目标基本实现，审批效率提高65%。市政务服务“网上办”事项达到92%，公共资源交易平台初步建成。文化、商务行政执法改革基本完成。驻连部队调整改革顺利推进，军民融合逐步深化。243家行业协会商会与行政机关脱钩。诚信连云港建设得到加强。农村土地确权颁证率达90%，试点土地经营权抵押租赁。供销社改革扎实推进。国有林场改革基本完成。国家创新型城市建设有序推进，国家知识产权试点城市建设通过验收。新增国家高新技术企业49家，国家工程技术研究中心、企业技术中心达到9家。国家重大科技基础设施“高效低碳燃气轮机试验装置”获批建设。获评国家专利优秀奖4个，正大天晴入围国家科技进步二等奖，豪森药业获省长质量奖，康缘等四大药企进入中国医药研发最佳工业20强。引进国家千人计划专家5人、领军人才68人。

五是生态文明建设取得新成效。深入落实战略环评成果转化，全国率先开展“三线一单”管理试点。深入开展“263”专项行动，减少煤炭消费24万吨，整治燃煤小锅炉1580台，有效控制重点行业挥发性有机物，深入推进化工园区环境整治，空气质量优良天数跃居全省第一。全面落实“河长制”，试点“湾长制”，21条入海河流纳入环境监测，大浦等5个污水处理厂提标改造，东盐河等5条黑臭水体整治完成，禁养区规模以上畜禽养殖场全部关闭搬迁。实施交通干线环境综合整治，“三大生态廊道”建设有序推进，完成秦山岛等一批生态修复工程，新增造林面积10万亩，林木覆盖率28.2%。省级以上生态县区实现全覆盖。

六是社会事业迈上新台阶。教育现代化建设水平保持苏北领先，“云海在线”上线运行，新建中小学、幼儿园33所，改造中小学校舍49万平方米，招生秩序得到规范，新增省级职教实训基地4个，淮海工学院创建江苏海洋大学列入国家“十三五”高校设置规划。市第一人民医院新院区投用。灌云县获评全国中医药工作先进县。医药卫生体制改革扎实推进，基层首诊率达72.4%，家庭医生签约率33.4%。居民电子健康卡全国首发。完成市文化馆、美术馆改造，市档案馆新馆启用，建成市图书馆分馆30个、基层综合文化服务中心680个，开展文化惠民活动2万场次。《辣妈犟爸》等3件作品获省“五个一工程”奖，4件作品获省文华奖。非遗传承和文物保护得到加强。新建改造城乡体育设施72处，城市“10分钟健身圈”进一步完善。连云港手机台上线运营。成功举办连云港之夏、西游记文化节、徐圩国际马拉松等活动。

七是群众幸福指数得到新提升。50件实事较好完成。实施就业创业富民工程，完成培训24.3万人次，城镇新增就业6.3万人，自主创业1.5万人，创业担保基金惠及1073人次。完成城乡居民医保制度整合，住院合规费用报销比例达到70%。城乡居民基础养老金持续增长。每千名老人拥有养老床位37张。城乡低保标准分别增长5.3%和10.1%。残疾人两项补贴覆盖6.3万人。发放困难学生补助1.02亿元。3568户困难家庭住房问题得到解决。10万低收入人口脱贫，50个经济薄弱村达到新“八有”标准。新增省级“民主法治示范村（社区）”70个。完成村（居）委会换届选举。阳光信访深入推进。扎实开展隐患排查和社会安全治理，群众安全感进一步提升。食品药品监管得到加强。各类社会组织作用有效发挥。民族宗教、外事侨务、机关事务、应急管理、地震气象、邮政通讯、对台工作取得新进步，统计、物价、民防、仲裁、地方志等工作得到新加强，妇女儿童、青少年、老龄、慈善、双拥、人民武装、国防动员、关心下一代、红十字等工作取得新发展。

过去一年，我们坚持依法行政，自觉接受人大法律监督、工作监督和政协民主监督，共办理人大代表建议230件、政协委员提案337件，办复率、满意率100%。严格落实中央八项规定，着力解决“四风”问题。加强行政监察和审计监督，开展政务公开与政风评议，落实党风廉政建设“一岗双责”，进一步转职能、改作风，“五型”政府建设得到加强。

各位代表，过去一年成绩来之不易，这是省委省政府和市委正确领导的结果，是全市人民团结奋斗的结果。在此，我代表市人民政府，向全市人民，向各位人大代表、政协委员，向各民主党派、工商联、人民团体和社会各界人士，向驻连部队、武警官兵、公安民警，向为连云港发展作出贡献的驻连部省属单位，向所有关心支持连云港的海内外朋友，表示衷心感谢和崇高敬意！

在肯定成绩的同时，我们也清醒认识到：经济总量小、产业层次低，发展不充分是我们面临的最大矛盾；公共服务供给不足，民生保障存在短板，社会事业不平衡问题相对突出；生态环境治理任务艰巨，安全生产领域矛盾凸显；政府自身建设还存在薄弱环节，工作作风有待进一步改进。对此，我们将坚持问题导向，系统谋划，综合施策，切实加以解决。

2018年主要任务

2018年是贯彻落实党的十九大精神的开局之年，是改革开放40周年，是决胜全面建成小康社会、实施“十三五”规划承上启下的关键之年。做好今年工作，意义重大。

今年政府工作总体要求是：全面贯彻党的十九大精神，以习近平新时代中国特色社会主义思想为指导，牢固树立新发展理念，紧扣社会主要矛盾变化，按照高质量发展要求，以深化供给侧结构性改革为主线，坚持稳中求进、好中求快，深入实施五大战略，统筹做好稳增长、促改革、调结构、惠民生、优生态、防风险各项工作，持续推进“两聚一高”新实践，努力打造“一带一路”战略支点，奋力开创“强富美高”连云港建设新局面。

全市经济社会发展主要预期目标是：地区生产总值增长7%以上，一般公共预算收入增长7.5%，规模以上固定资产投资增长8%，社会消费品零售总额增长10%，实际利用外资6亿美元，城乡居民收入分别增长8%和8.5%，居民消费价格指数、节能减排完成省定目标。

围绕以上目标任务，重点做好以下工作：

（一）坚定不移推进产业强市，更高质量提升发展水平。瞄准产业链、价值链高端，狠抓项目、狠抓投入，力争全市产业投资突破千亿，培育一批带动力强的优质项目，做大一批成长性好的骨干企业，着力构建现代产业体系。

大力发展临港产业。放大港口综合优势，加快培育千亿级企业集群。坚守环保安全、工艺设备、投入产出、品质品牌四个“世界一流”标准，高水平建设国家级石化产业基地，重点抓好盛虹石化、新海石化、东华能源、中化国际、卫星石化、益海盐化等重大项目落地建设。加快延伸产业链，形成集聚效应，带动临港产业迅速兴起。遵循低碳、循环、集聚发展模式，加快推动钢铁、化工等产业升级改造，提升可持续发展水平。

快速壮大优势产业。推进恒瑞、正大天晴、长生生物等新项目建设，做大做强医药产业。加快中复神鹰T1000碳纤维产业化，推进中复碳芯、奥神新材料等企业提升核心竞争力。田湾核电3、4号机组投入运营，正崴新能源、华能海上风电等加快建设。开展“三千”技改行动，提升食品、纺织、装备制造等传统产业，工业技改投资增长10%。深入推进企业培育“三大行动计划”，加强政银企对接，助推中小微企业发展。推进“两化”融合，强化智能支撑，研发核心技术，加快连云港制造向连云港创造迈进。

突破发展海洋经济。加快国家海洋经济示范区建设，推进海洋经济普查，统筹海洋深度开发和海岸高效利用，加速资源优势向经济优势转化。持续推进海州湾海洋牧场建设，推广近海新兴养殖模式，支持发展深远海渔业。依托海福特等龙头企业加快发展海洋食品工业，年内加工能力达到45万吨。积极培育海工装备、生物医药、海水利用等新兴产业，发展邮轮经济、海上运动等新兴业态。提升海洋文化体验，深度开发徐福文化、淮盐文化品牌。

加快发展现代服务业。把现代服务业作为推动经济转型升级的重要引擎。以重点项目和载体为抓手，推动生产性服务业提速发展。培育壮大连农云商等平台，做强天马网络等电商基地，推动线上线下融合发展。筹建市综合金融服务平台和股权交易中心，健全融资担保体系，引进金融机构3家以上。新增社会融资600亿元。优化供给结构，推动生活性服务业高端发展。高标准规划建设高铁商圈，着力打造集交通集散、旅游服务、精品酒店、特色购物于一体的城市新门户。推进港华综合体等商贸项目建设。加快文化创意、专业设计等业态发展。连云港大剧院试运营。实施旅游强市三年行动计划，加快构建“一带三轴”旅游布局，打造五大特色产品体系，推动传统旅游向现代休闲度假旅游转变。

（二）推进双向开放，更高站位建设“一带一路”战略支点。发挥双向开放优势，深化“港航路园”合作，打造标杆和示范项目，谋划申报自由贸易港试点，不断增创开放发展新优势。

完善港口功能。围绕枢纽港、产业港、贸易港、物流港的定位，加快港口转型升级。推进30万吨级航道二期工程建设，基本建成徐圩4区围堤工程，完善液体化工码头、港区公共管廊等配套设施。实施赣榆港区10万吨级航道项目。完成灌河港区燕尾三期工程水工主体。加快徐圩、赣榆港区铁路专用线建设。基本完成灌河航道整治，推进徐宿连航道前期工作。新增非洲远洋干线，加密集装箱航班航线。争取建设中铁集装箱中心站。提升口岸服务水平，建成国际贸易“单一窗口”和口岸监管信息平台。力争综合保税区获批运行、两翼口岸正式开放。

提升“两基地”建设运营水平。推进中哈物流基地散粮筒仓、保税仓库等二期项目，开发粮食、汽车整车等运输业务，力争运量突破50万标箱。提高“霍尔果斯-东门”无水港运营效率，推动东西口岸协同发展。持续推进上合物流园建设，专用铁路主体基本完工，加快建设中外运上合物流中心、大宗商品交易中心，“一带一路”商贸展示中心、矿石精选中心投入使用。加密中欧班列，扩大运行规模，年开行800列。拓展周边市场，扩大海河联运。

深入推进开放合作。连云港发展优势在开放，必须以大开放拓展发展新空间，以大招商汇聚发展新动能，以招大商形成发展新依托。狠抓产业链招商，强攻突破源头性重大产业项目，带动下游企业蓬勃发展。推进以商引商，服务好现有企业，用好口碑、好声誉吸引客商。加强专业招商队伍建设，强化精准招商，精准遴选、精准对接，切实提高招商成效。注重招商引资与招才引智相结合，促进人才、项目、产业互动并进。充分发挥连博会、国际医药技术大会等平台作用，放大连云港论坛综合效应，促进更多项目落地见效。推进优进优出，实现外贸增长5%，服务贸易增长10%。鼓励更多企业“走出去”。

（三）完善功能提升品质，更富魅力建设海港城市。强化以人为中心的城市建设理念，巩固“干净、方便、顺畅、安全”连云港建设成果，推进“生态、智慧、健康、文明”连云港建设。

优化城市功能。坚持规划引领，统筹城市空间和要素配置，合理确定开发时序，推进城区组团发展。赣榆城区主动融合对接，海州城区加快均衡发展，连云城区打造滨海特色。各功能板块强化产业定位，实现错位发展。高标准推进县城建设，强化县域中心功能。连盐铁路通车运营，连青铁路建成，连徐高铁、连淮扬镇铁路快速推进。加快连淮高速、204国道、236省道等干线公路改扩建。开工建设新机场。建设赣榆至海州快速通道，实施人民路快速化改造，打通陇海西路等“断头路”。有序实施地下综合管廊和海绵城市建设。提升城市防洪标准。实施市殡仪馆搬迁。新改建供水、燃气等管道30公里，建成110千伏及以上输变电工程33个，改造低压接线25万户。实现光纤宽带和4G网络全覆盖。

提升城市品位。坚持城市内涵发展，推动公共供给的体系化、均衡化、品质化。加强重点片区、重要节点景观设计，打造“显山、露水、见海、融绿”城市天际线。启动园博园建设，推进瀛洲公园等18个园林绿化项目，建设一批小游园、小绿地、小广场，合理配置休闲座椅、健身器材等功能性设施，打造设施齐全、服务完善的城市公共空间。扎实推进50个老旧小区改造，棚户区改造新开工2万套。加强城市地标和主要街道标识设计，高品位建设一批城市雕塑、文化小品，进一步提升城市家具标准化水平。

加强精细管理。巩固提升创卫成果，突出细致、深入、规范，建立目标量化、标准细化、职责更加具体化的城市管理模式。加强环境卫生管理，强化环卫设施建设，继续推进“厕所革命”，实行垃圾分类处理。持续开展市容市貌专项整治，推进城市管理法制化、规范化。提高物业服务覆盖率，严格物业管理标准，创建市级以上示范物管项目35个。加快新型智慧城市建设，用好大数据，提高城市管理的智能化、科学化水平。

（四）落实乡村振兴战略，更大力度做好“三农”工作。按照产业兴旺、生态宜居、乡风文明、治理有效、生活富裕总要求，发展农村生产力，有效增加农民收入，加快推进农业农村现代化。

发展现代农业。深入推进农业供给侧结构性改革，加快构建现代农业产业体系。优化调整农业结构，农牧渔结合，种养加一体，推进一产接二连三，实现农产品供给向更加优质生态高效转变。增强农业综合生产能力，落实粮食安全责任制，建设优质稻米产业基地20个，新增高效设施农业3万亩、节水灌溉4万亩、现代渔业1万亩。提高农业经营规模化水平，新增市级以上龙头企业30家，放大“连天下”品牌效应，新增“三品”品牌100个以上。推进国家农产品质量安全市创建，创成省级农产品质量安全县1个。启动国家农业对外开放合作试验区建设，农产品出口保持全省领先。新建储粮仓容5万吨。农机化综合水平达81.5%。

推动农村改革。健全城乡融合发展体制机制，推动资本、技术、人才等要素向乡村流动。强化土地承包经营权确权登记成果运用，采取土地入股、托管、代耕代种等形式，让农户承包权稳下去、经营权活起来。深化农村集体产权制度改革，多途径发展壮大集体经济，推进400个村集体资产股份制改革，农村产权交易额保持10亿元以上。加快发展农村电子商务，培育家庭、集体、合作社、企业等多元经营主体，更多渠道促进农民增收。

打造宜居环境。完善农村公共服务基础配套，优化功能布局，建设美丽宜居乡村。继续实施镇村道路提升行动，新建改造600公里。持续开展农村环境综合整治，推进农村“四改”，加强“四旁”绿化，新建省级绿化示范村40个，疏浚沟渠河道460公里。坚持分类指导，统筹推进特色小镇、特色田园乡村建设。传承乡土文化，培育文明乡风，健全自治、法治、德治相结合的乡村治理体系，打造充满活力、和谐有序的乡村社会。

（五）深化改革创新，更加有效激发内生动力。坚持问题导向、发展取向，加大重点领域和关键环节的改革力度，努力在体制机制和科技创新等方面实现新的突破。

深化供给侧结构性改革。优化供给结构，扩大有效和中高端供给。突出重点、压实责任，在“破”“立”“降”上下更大功夫。继续推进“三去一降一补”，年内淘汰整合冶金、化工企业18家，化解船舶产能30万吨。加强土地利用计划管理，把握城区土地供给节奏，大力发展住房租赁市场，扩大住房公积金覆盖面。深化产权制度和要素市场化配置改革，推动生产要素从低质低效向优质高效领域流动。采取更加得力措施，帮助企业进一步减费降本。针对薄弱环节，补齐民生、安全、环保等短板。

深化“放管服”改革。全面实施市场准入负面清单，压减生产许可事项。细化政府部门职责，制定“双随机”抽查细则。推进数字政府建设，建成市大数据中心二期和政务数据库。完善“互联网+政务服务”体系，实行政务服务“一张网”，让数据多跑路、群众少跑腿。建设网上“中介超市”，规范中介管理。深化“不见面”改革，企业投资建设项目全面推行代办制，打造“审批事项更少、办事效率更高、发展环境更优”的政务生态系统。

深化重点领域改革。强化政府性债务管理，有效防范财政金融风险。推进国资国企改革，深化国有资本投资运营公司综合性改革，支持国有企业压缩管理层级，整合同质同业子公司，完成5家企业混合所有制改革，基本完成“三供一业”分离移交和企业办社会改制。推进普惠金融试验区建设，加大股权融资力度。深化园区体制改革，集中精力抓好经济管理和投资服务。完成企事业单位公车改革、市县环保监测监察执法机构改革。深化户籍制度改革，进一步放宽租赁房屋落户条件。继续推进经济发达镇行政管理体制改革试点。支持军队改革，推进军民融合发展。

深入推进科技创新。实施创新驱动战略，推进创新型城市建设。落实省科技创新“40条”扶持政策，突出企业主体地位，打造产业技术协同创新联盟，重点培育省创新型领军企业2家、省级首台套及关键部件产品2个，实施省级以上重大科技创新与成果转化项目30项，新增国家高新技术企业30家，新建研发机构20家。推进质量强市，争创全国知名品牌示范区1个。万人发明专利拥有量突破5.5件。加强企业家队伍建设，强化人才支撑，新引进国家“千人计划”等尖端人才3名、省“双创计划”人才15名。

（六）坚决守住生态底线，更实举措建设美丽家园。绿水青山就是金山银山，坚定不移地走生态优先、绿色发展之路，坚持源头严防、过程严管、后果严惩，坚决呵护好港城人民的美好家园，实现美丽与发展共赢。

大力推动绿色发展。实施绿色制造行动计划，积极发展“生态+”经济，推动生产方式绿色化转型。严格落实“三线一单”，强化环境硬约束。严把“产业导向、项目准入、节能减排”三个关口，提高准入门槛。实行最严格的水资源管理、节约用地制度，确保万元GDP能耗、水耗“双下降”。完善环保基础设施，市级以上开发园区规划环评全部通过审查。深入实施农业绿色发展五大行动，增强可持续发展能力。倡导低碳生活方式，创建节约型社会。

全面整治突出问题。持续推进“263”专项行动，坚决打好污染防治攻坚战。深入实施“水十条”，推进清水绕城，完成市区黑臭水体整治，消除劣五类水质考核断面。县级以上饮用水源地全部达标。持续实施空气质量提升行动，做好重污染天气预警应对，空气优良天数保持全省领先。全面落实“土十条”，推广秸秆还田、保护性耕作，开展罗盖特等地块治理修复。完成40家企业清洁化改造，全面启动化工园区创建省级生态工业园区。

加快推进生态文明建设。启动大规模国土绿化行动，开展国家森林城市创建，打造徐新公路等一批森林大道，完成绿化造林15万亩，云门寺采石场等5处破损山体整治复绿。推进临洪河口湿地公园建设，自然湿地保护率达到48%。开展生物多样性本底调查。争取国家“蓝色海湾”“生态岛礁”工程，推进海岸线整治修复。创建生态文明建设示范区，建成10个省级示范乡镇和5个示范村。深入实施“河长制”，扎实推进“湾长制”。开展领导干部自然资源资产离任审计，强化生态文明绩效考核与责任追究。

（七）全面发展社会事业，提供更加优质均衡的公共服务。牢牢把握人民群众对美好生活的向往，抓重点、补短板、强弱项，推进基本公共服务标准化、均等化，提高服务能力和共享水平。

努力办好人民满意教育。把教育摆在优先发展位置，东海、海州、连云、市开发区完成省教育现代化建设任务。新建幼儿园26所，规范提升学前教育。新改建中小学16所，75%义务教育学校达到省定现代化办学标准。切实解决“大班额”“择校热”等问题，开展小学弹性离校试点，努力办好家门口每一所学校。高中教育提升品质，职业教育特色发展，高等教育内涵提升，支持淮海工学院创建江苏海洋大学。国家教育信息化达标率85%。招收中亚留学生100名。市特教中心新校区建成投用。加强师德师风建设，提升教师队伍综合素质。

扎实推进文化建设。践行社会主义核心价值观，持续推进文明城市创建。实施文艺精品工程，深化“书香连云港”建设。推进文化重点项目，建成370个基层综合性文化服务中心。持续开展文化惠民活动，组织万场电影千场戏展映展演。加强东海孝妇传说等非遗项目开发利用，开展南城古镇保护修复，做好宿城大竹园村等传统村落的保护开发。申报国家历史文化名城。建设市数字档案馆。办好连云港之夏、西游记文化节。

加强健康连云港建设。延伸扩大创卫成果，力争三县创成国家卫生县城，省级以上卫生乡镇达50%。健全现代医院管理制度，完善分级诊疗和家庭医生制度，探索医保支付方式改革。构建紧密型医联体，促进医疗资源下沉。加强基层医疗卫生队伍建设。居民电子健康档案建档率达80%以上。推进省海滨医院、市康复医院项目，加快市第二人民医院西院区、市妇幼保健中心建设。实施“健康宝贝工程”。建成市青少年足球基地，全民健身中心加快建设，举办市首届全民健身运动会。

（八）坚持民生优先，让人民群众有更多获得感幸福感。坚持以人民为中心，始终把群众利益摆在首位，加强民生保障，扎实办好实事，让港城发展更有温度、百姓幸福更有质感。

促进居民收入持续增长。扎实推进富民增收六大行动，实施“创响港城”计划，扶持引导大学生、退役士兵、农民工等自主创业万人以上，创业带动就业4万人。开展职业培训22万人次，加强就业服务，解决好招工难与高质量就业难矛盾，新增城镇就业6万人，失业人员再就业1.6万人，城镇零就业家庭动态为零。推动企业普遍建立工资集体协商制度，适时提高最低工资和低收入人群补贴标准。推动产业“下乡入户”，促进农民家门口就业。

提高社会保障水平。坚持保基本、兜底线、促公平、可持续，进一步织密社保网络。推进重点人群扩面参保，执行城乡居民统一的医保制度，完成退休人员养老金调整，城乡居民基础养老金增长8%。稳步提高城乡低保标准和残疾人保障水平，实现“救急难”全覆盖。分配公租房600套，对老旧小区困难家庭物管费用实行财政补贴。新建标准化居家养老服务中心30个。推进公益性菜市场建设，完善价格补贴机制，保障困难群众基本生活。

精准扶贫脱贫。瞄准贫困群体和重点片区，因村因户因人具体施策，年内4万人脱贫、50个经济薄弱村达到新“八有”标准。实施产业扶贫，扶持薄弱村发展特色经济。开展交通、水利等专项扶贫，实施石梁河、沂河淌片区路网和赣榆丘陵节水灌溉项目改造。推行“互联网+”扶贫，创建市级电商扶贫示范村10个。加大金融扶持力度，发放扶贫贷款3亿元以上。积极开展教育、医疗、科技扶贫，重点解决好因病因残致贫返贫问题。强化政策扶贫，加强兜底保障。实施扶贫阳光监管，确保每一分钱都用在刀刃上，绝不让一户一人在小康路上掉队。

加强社会治理创新。深入推进依法治市，完善治安防控体系，创建“最安全城市”。实施“雪亮工程”，建设反电信网络诈骗犯罪中心。扎实开展“七五”普法，创建省级民主法治示范村（社区）65个。深化“三社联动”机制，全面推广“一委三会”社区治理。深刻汲取灌南“12·9”重大事故教训，坚持最严格的安全标准，以铁的纪律、铁的手腕落实铁的责任，坚决遏制重特大事故发生。提升12345政府热线服务水平。加强法治信访建设。规范市场秩序。强化食品药品监管。完善应急广播体系。推进诚信连云港建设。加强社会组织管理。更加关爱老人、妇女儿童、青少年和残疾人，进一步做好民族宗教、外事侨务、地震、民防、气象、科普、地方志、关心下一代、红十字、慈善、援疆等工作。

各位代表，今年我们将继续加大力度，从百姓最关心的事情入手，聚焦十大重点领域，扎实办好关注度高、惠及面广、当年见成效的50件民生实事，让广大群众共享更多更好的发展成果。

加强政府自身建设

新时代要有新气象，更要有新作为。我们要深入贯彻党的十九大精神，学思践悟习近平新时代中国特色社会主义思想，牢固树立“四个意识”，进一步加强自身建设，提升执行力和服务效能，努力打造人民满意的“五型”政府。

强化政府执行能力。一分部署，九分落实。政府工作既需求真务实、系统谋划，更要埋头苦干、真抓实干、敢抓敢干、善谋善干。雷厉风行抓落实，强化立说立行，推行马上就办。勇于担当抓落实，勇闯深水区，敢啃硬骨头，问题面前不回避，困难面前不退缩。优化机制抓落实，强化督查考核，将决策部署与督办推进同安排、同部署，确保各项工作早落实、快见效。

切实提升服务效能。准确把握企业需求、基层呼声和群众期盼，强化服务意识，提高服务本领，提升服务水平。适应高质量发展新要求，加强经济运行新趋势、新特征、新变化的研判，增强服务发展的能力。努力营造低成本、更便利、公正公平的发展环境，真正做到有求予助、无事不扰、贴心服务。深入基层，深入群众，了解社情民意，把群众的期盼作为我们工作的第一目标，把基层的呼声作为工作的第一信号，把群众的评判作为工作的第一标准，以优质的服务赢得群众的口碑。

深化法治政府建设。把政府工作全面纳入法治轨道，健全科学民主决策机制，完善公众参与、专家论证、风险评估、合法性审查、集体讨论决定等重大行政决策程序。执行重大事项向市人大常委会报告制度。对民生重要事项开展听证，规章草案公开征求意见。加强政务公开和政策解读，让行政权力依法规范运行。

加强廉洁政府建设。坚持把纪律挺在前面，强化权力制约监督，坚定不移推进政府系统廉政建设和反腐败工作。牢记作风建设永远在路上，从严落实“八项规定”，重拳整治“四风”问题新形式新表现。扎实开展政府系统“不忘初心、牢记使命”主题教育活动。加强对公共资金、公共资源交易、国有资产资本的审计监督。自觉接受人大及其常委会的法律监督、工作监督和政协的民主监督，主动接受社会公众监督和舆论监督。

各位代表，实干托起梦想，奋斗铸就辉煌。立足新时代，展望新征程，我们深感责任重大，使命光荣。让我们高举习近平新时代中国特色社会主义思想伟大旗帜，紧密团结在以习近平同志为核心的党中央周围，在市委的坚强领导下，坚定信心、开拓进取，同心同德、真抓实干，为高水平全面建成小康社会、建设“强富美高”新港城而努力奋斗！
